# Circulating plasma exosomal long non-coding RNAs LINC00265, LINC00467, UCA1, and SNHG1 as biomarkers for diagnosis and treatment monitoring of acute myeloid leukemia

**DOI:** 10.3389/fonc.2022.1033143

**Published:** 2022-10-07

**Authors:** Qiaoling Xiao, Can Lin, Meixi Peng, Jun Ren, Yipei Jing, Li Lei, Yonghong Tao, Junpeng Huang, Jing Yang, Minghui Sun, Jing Wu, Zailin Yang, Zesong Yang, Ling Zhang

**Affiliations:** ^1^ Key Laboratory of Laboratory Medical Diagnostics Designated by the Ministry of Education, School of Laboratory Medicine, Chongqing Medical University, Chongqing, China; ^2^ Department of Hematology, The First Affiliated Hospital of Chongqing Medical University, Chongqing, China; ^3^ Department of Hematology-Oncology, Chongqing Key Laboratory of Translational Research for Cancer Metastasis and Individualized Treatment, Chongqing University Cancer Hospital, Chongqing, China

**Keywords:** acute myeloid leukemia, biomarker, exosome, long non-coding RNA, LINC00265, LINC00467, UCA1, SNHG1

## Abstract

Exosomal long non-coding RNAs (lncRNAs) have emerged as a cell-free biomarker for clinical evaluation of cancers. However, the potential clinical applications of exosomal lncRNAs in acute myeloid leukemia (AML) remain unclear. Herein, we attempted to identify plasma exosomal lncRNAs as prospective biomarkers for AML. In this study, plasma exosomes were first successfully extracted from AML patients and healthy donors (HD). Subsequently, the downregulated plasma exosomal lncRNAs (LINC00265, LINC00467, and UCA1) and the upregulated plasma exosomal lncRNA (SNHG1) were identified in AML patients (n=65) compared to HD (n=20). Notably, individual exosomal LINC00265, LINC00467, UCA1, or SNHG1 had a capability for discriminating AML patients from HD, and their combination displayed better efficiency. Furthermore, exosomal LINC00265 and LINC00467 were increased expressed in patients achieving complete remission after chemotherapy. Importantly, there was upregulation of exosomal LINC00265 and downregulation of exosomal SNHG1 upon allogeneic hematopoietic stem cell transplantation. Additionally, these lncRNAs were high stability in plasma exosomes. Exosomal LINC00265, LINC00467, UCA1, and SNHG1 may act as promising cell-free biomarkers for AML diagnosis and treatment monitoring and provide a new frontier of liquid biopsy for this type of cancer.

## Introduction

Acute myeloid leukemia (AML) is an aggressive and molecularly heterogeneous hematologic malignancy characterized by clonal differentiation arrest and uncontrolled proliferation of myeloid blasts in the bone marrow (1). As the most common form of acute leukemia in adults, AML has a worldwide incidence of approximately 5.4 per 100,000 per year (2). Nowadays, the diagnosis of AML is based on the conjoint assays of morphology, immunophenotype, cytogenetics, and molecular genetics, which requires the presence of circulating blasts in either a blood or a bone marrow sample (3). However, early detection of this disease remains a major challenge, due to the late dissemination of leukemic blasts to the peripheral blood and symptoms appearing late. Until recently, treatment options for AML in the clinic mainly involve cytotoxic chemotherapy, targeted therapy, and allogeneic hematopoietic stem cell transplantation (allo-HSCT) (4). Although the majority of AML patients respond to the initial standard induction chemotherapy, primary refractory and relapse lead to lower long-term survival rates (5). Therefore, it is particularly important to monitor treatment efficacy in real-time and make appropriate therapeutic judgments. It has been well acknowledged that the treatment monitoring of AML mainly depends on comprehensive cellular analyses, which is complicated by the prompt clearance of circulating leukemic blasts during therapy. Hence, the discovery of reliable cell-free biomarkers for early detection and treatment monitoring of AML has been urgently needed. Our previous study revealed that circulating DNA in plasma might be a prospective diagnostic and disease tracking predictor for acute leukemia (6), whereas plasma DNA reveals information from mostly dying cells and failed to detect early lesions (7). Herein, we attempt to identify novel cell-free biomarkers for AML timely diagnosis and surveillance.

Exosomes are a subset of extracellular vesicles with lipid bilayer membranes, ranging from 30 to 150 nm in diameter (8). It is known that exosomes are released continuously by a variety of living cells and are present in human biofluids such as saliva, blood, and urine (9–11). In fact, exosomes can stably carry abundant parental cell-derived bioactive molecules (nucleic acids, proteins, and lipids) due to the bilayer membrane structure and provide real-time signals from living cells (12). Thus, circulating exosomes and especially their contained cargoes have gained increased attention in cancer liquid biopsy, highlighting the potential as biomarkers for cancer diagnosis, progression monitoring, and prognosis prediction (12). Notably, the extracellular vesicle concentration was found to elevate in the plasma of the AML patient at diagnosis and remained increased even at complete remission after chemotherapy (13). More importantly, in AML cell line Molm-14 and patient-derived xenografted murine models, circulating AML-derived extracellular vesicles were demonstrated to spread into the peripheral blood ahead of the leukemic blasts and associate with disease burden (14). These reports suggest that extracellular vesicles have a great promise to act as cell-free indicators for AML early diagnosis and disease tracking during therapy. Recently, the ongoing development of sequencing technologies has permitted an increase in the number of newly discovered long non-coding RNAs (lncRNAs). LncRNAs represent a kind of non-coding RNAs with limited protein-coding capacity longer than 200 nucleotides (15). Emerging evidence has corroborated that lncRNAs play important roles in leukemogenesis. Long intergenic non-protein coding RNA 265 (LINC00265) was highly expressed in the bone marrow and serum of AML patients and related to AML diagnosis (16). Furthermore, LINC00265 inhibited leukemic cell apoptosis by inducing autophagy (17). Silencing of long intergenic non-protein coding RNA 467 (LINC00467) suppressed the malignant phenotypes of AML cells (18). Liang et al. (19) reported lncRNA urothelial cancer associated 1 (UCA1) knockdown attenuated proliferation and accelerated apoptosis in AML cells. In addition, the upregulation of lncRNA small nucleolar RNA host gene 1 (SNHG1) was observed and SNHG1 served as an oncogene in AML (20). Besides, lncRNA prostate cancer associated transcript 18 (PCAT18) showed higher expression in AML samples and promoted leukemic cell proliferation (21). LncRNAs enclosed in exosomes are prevented from ribonuclease-mediated degradation and stably exist in body fluids (22). Accumulating studies have demonstrated that exosomal lncRNAs exert important functions in carcinogenesis and development. Zang et al. (23) reported that exosomal lncRNA UFC1 could facilitate tumor cell growth, migration, and invasion in non-small cell lung cancer (NSCLC). In another study, exosome-transmitted lncRNA Sox2ot promoted the epithelial-mesenchymal transition process and induced stem cell-like properties of pancreatic ductal adenocarcinoma cells (24). Additionally, LINC00461 was upregulated in mesenchymal stromal cell (MSC)-derived exosomes and further enhanced multiple myeloma cell proliferation (25). Based on essential roles in cancers, exosomal lncRNAs have become of tremendous interest in biomarker research. From recent studies, tumor-derived exosomal lncRNA GAS5 was reported to be highly sensitive to early-stage NSCLC (26). Sedlarikova et al. (27) identified serum exosomal lncRNA PRINS as a novel biomarker for multiple myeloma diagnosis. Besides, serum exosomal lncRNA aHIF might be an unfavorable prognostic factor of epithelial ovarian cancer (28). Our previous study also suggested that exosomal lncRNAs TBILA and AGAP2-AS1 exhibited powerful diagnostic efficiencies in NSCLC patients with different tumor pathologic subtypes and early stages (29). Nevertheless, the potential clinical utility of exosomal lncRNAs in AML has not been reported yet.

In the present study, plasma exosomes were first successfully isolated from the AML patient and the healthy donor (HD). Subsequently, the decreased plasma exosomal lncRNAs (LINC00265, LINC00467, and UCA1) and the increased plasma exosomal lncRNA (SNHG1) were identified in AML patients (n=65) compared to HD (n=20). Of note, individual exosomal LINC00265, LINC00467, UCA1, or SNHG1 had a capability for distinguishing AML patients from HD, and the combination of these four exosomal lncRNAs exhibited the most powerful diagnostic accuracy. Furthermore, when the patients achieved complete remission but not non-remission or partial remission after the standard induction chemotherapy, the level of exosomal LINC00265 and LINC00467 elevated. More importantly, there was upregulation of the exosomal LINC00265 level and downregulation of the exosomal SNHG1 level undergoing the allo-HSCT treatment. Additionally, these lncRNAs were high stability in plasma exosomes. Our observations prove that exosomal LINC00265, LINC00467, UCA1, and SNHG1 may act as novel cell-free indicators for AML diagnosis and treatment monitoring and provide a new frontier of liquid biopsy for this type of cancer.

## Materials and methods

### Study subjects and clinical samples

A total of 65 patients with newly diagnosed acute myeloid leukemia (AML) and 20 healthy donors (HD) were enrolled in this study from December 2020 to August 2021 at the First Affiliated Hospital of Chongqing Medical University. Under the 2019 National Comprehensive Cancer Network (NCCN) guideline of AML (30), all the patients were diagnosed with AML based on the conjoint analyses of morphology, immunophenotype, cytogenetics, and molecular genetics. On the basis of the French-American-British (FAB) classification of AML (31, 32), the patients were divided into six subtypes including M0 (minimally differentiated acute myeloid leukemia), M1 (acute myeloblastic leukemia without maturation), M2 (acute myeloblastic leukemia with maturation), M3 (acute promyelocytic leukemia), M4 (acute myelomonocytic leukemia), and M5 (acute monoblastic or monocytic leukemia). Meanwhile, the cases were separated into three risk classifications including favorable, intermediate, and adverse according to the 2017 European LeukemiaNet (ELN) risk stratification guideline based on cytogenetic abnormalities and genetic mutations (33). Consistent with the 2019 NCCN guideline, all the AML participants received the recommended treatment regimens, and remission responses to chemotherapy including non-remission (NR), partial remission (PR), complete remission (CR), and disease recurrence of patients were assessed. The AML patients co-existing with other types of tumors or subjected to chemotherapy or radiotherapy before blood collection were excluded. Additionally, 12 plasma samples from enrolled AML patients at the first CR stage after the standard induction chemotherapy were collected. Before and after allogeneic hematopoietic stem cell transplantation (allo-HSCT), 12 paired plasma samples from enrolled AML patients were collected. Besides, the healthy donors who underwent routine physical examinations and showed no signs of disease were recruited as controls. The healthy individuals were sex- and age-matched to AML patients. This research was approved by the Ethics Committee of Chongqing Medical University. The experiments were conducted in accordance with the Helsinki Declaration. Written informed consent was obtained from all the subjects for study purposes. Details of the clinical characteristics of all the enrolled study subjects are presented in **Table 1**.

**Table 1:** Clinical characteristics of study subjects.

**Categories**	**Healthy donors** **(n = 20)**	**AML patients** **(n = 65)**	***P*-value**
Gender, No. (%)			
Female	9 (45.00)	34 (52.31)	0.5676
Male	11 (55.00)	31 (47.69)	
Age (years), mean ± SD	47.50 ± 9.69	52.00 ± 16.41	0.6510
Peripheral blood, median (IQR)			
WBC count (×10^9^/L)		10.97 (3.14-49.15)	
PLT count (×10^9^/L)		37.00 (13.50-73.50)	
Hb level (g/L)		78.00 (59.50-94.50)	
LDH level (U/L)		656.00 (394.00-964.50)	
Bone marrow blasts (%), median (IQR)		65.00 (30.78-86.36)	

AML, acute myeloid leukemia; WBC, white blood cell; PLT, platelet; Hb, hemoglobin; LDH, lactate dehydrogenase; SD, standard deviation; IQR, interquartile range. The chi-square test was used to compare differences in gender between two groups and determine P values. The Shapiro-Wilk normality test was performed to test normality, and then the unpaired Student’s t-test was used to compare differences in age between two groups and determine P values. P < 0.05 means statistically significant.

### Cell culture

Human AML cell line NB4 was purchased from American Type Culture Collection (ATCC, Manassas, VA, USA) and cultured in RPMI-1640 medium (Thermo Fisher Scientific, Waltham, MA, USA, #11875093) containing 10% fetal bovine serum (Thermo Fisher Scientific, #10091155). The mediums were supplemented with 1% Penicillin-Streptomycin solution (Beyotime, Shanghai, China, #C0222) to protect cells from potential contamination, and cells were incubated at 37°C in the presence of 5% CO2.

### Plasma exosome isolation

Peripheral blood specimens from all the participants were collected in vacuum blood tubes with ethylenediaminetetraacetic acid (EDTA) anticoagulant. Followed by a two-step centrifugation protocol (2,000 g at 4°C for 10 min; 10,000 g at 4°C for 30 min), plasma samples were acquired. To eliminate contaminating cell debris and large diameter extracellular vesicles, the supernatants were filtered through a 0.22 µm filter (Biosharp, Beijing, China, #BS-PES-22) and stored at -80°C until use. Exosomes were isolated from pretreated plasma samples using the exoRNeasy Midi Kit (QIAGEN, Dusseldorf, Germany, #77144) according to the manufacturer’s guidelines. In brief, 1 volume of plasma was mixed with 1 volume of binding buffer (XBP) and the sample/XBP mix was added onto the exoEasy spin column to bind exosomes to the spin column membrane. After centrifugation at 3, 000 g at room temperature (RT) for 1 min, the flow-through was discarded and 3.5 mL wash buffer (XWP) was added to the spin column to remove residual buffer by centrifugation at 3, 000 g at RT for 5 min. The flow-through and the collection tube were discarded, and then the spin column was transferred to a fresh collection tube. Finally, 400 μL elution buffer (XE) (QIAGEN, #76214) was added to the spin column to elute exosomes, followed by incubation at RT for 5 min and centrifugation at 500 g at RT for 5 min. The exosome suspension was collected and stored at -80°C for further study.

### Plasma exosome identification and quantification

The morphologies of isolated exosomes were visualized by transmission electron microscopy (TEM). Briefly, exosomes were loaded onto a carbon-coated 300 mesh copper grid (ProSciTech, Kirwan, Queensland, Australia). After drying at RT for 5 min, the grid was fixed with 2% glutaraldehyde in 0.1 M phosphate buffer (pH=7.4), and then stained with a drop of 2% uranyl acetate (Sigma-Aldrich, Burlington, MA, USA) at RT for 10 min. The morphologies of exosomes were observed by the JEM-1011 TEM (Hitachi, Tokyo, Japan).

The size distribution and concentration of exosomes were determined by nanoparticle tracking analysis (NTA). Isolated exosomes were resuspended in phosphate-buffered saline, and then injected into the ZetaView PMX 110 (Particle Metrix, Meerbusch, Germany). Particles were tracked and the size of particles was measured based on Brownian motion and the diffusion coefficient. Data were analyzed using the manufacturer’s software, ZetaView (Version 8.02.28).

The level of exosome marker proteins (CD63 and Alix) and non-exosomal protein (Calnexin) was detected by western blot analysis, as previously reported (34). In brief, Exosome suspensions were lysed in RIPA buffer (Beyotime, #P0013C) containing the protease inhibitor (Bimake, Houston, TX, USA, #B14001) on ice for 30 min. After centrifugation at 13,300 rpm at 4°C for 30 min, the supernatant was collected and quantified by the Enhanced BCA Protein Assay Kit (Beyotime, #P0010S), followed by boiling in 5×sodium dodecyl sulfate-polyacrylamide gel electrophoresis (SDS-PAGE) loading buffer (Beyotime, #P0015) for 10 min. Next, the isolated exosomal protein was separated by 12% SDS-PAGE and electro-transferred onto polyvinylidene fluoride membranes (Bio-Rad, Hercules, CA, USA, #1620177). The membranes were incubated with primary antibodies against CD63 (1:1000, Bimake, #A5177), Calnexin (1:1000, Cell Signaling Technology, Danvers, MA, USA, #2679), and Alix (1:500, Wanleibio, Shenyang, China, #WL03338) at 4°C overnight. Then, the membranes were covered with the secondary antibody (1:5000, Biosharp, #BL003A) at RT for 1 h, and the signals of labeled proteins were visualized using an enhanced chemiluminescence solution (Bio-Rad, #1705062).

### Plasma exosomal RNA extraction

RNA was extracted from plasma exosomes by the aforementioned exoRNeasy Midi Kit according to the manufacturer’s guidelines. Briefly, 700 μL QIAzol was added to the spin column membrane capturing exosomes or exosome suspensions to lyse the exosomes, and the lysate was incubated at RT for 5 min. Then, 90 μL chloroform was added to the lysate, and the mix was shaken vigorously for 15 s, followed by incubation at RT for 2 min. After centrifugation at 12,000 g at 4°C for 15 min, the upper aqueous phase was collected and mixed with 2 volumes of 100% ethanol. The mix was transferred to the RNeasy MinElute spin column and centrifuged at 8,000 g at RT for 15 s. The spin column was washed once with 700 μL Buffer RWT, and then twice with 500 μL Buffer RPE. After centrifugation at a full speed at RT for 5 min with the open lid, the spin column was added with 14 μL RNase-free water and stood for 1 min, followed by centrifugation at a full speed at RT for 1 min to elute the RNA for further analysis.

### Quantitative real-time polymerase chain reaction (qRT-PCR)

Plasma exosomal RNA was reversely transcribed into cDNA in 20 μL reaction volume by using PrimeScript RT Master Mix (Perfect Real Time) (Takara, Kyoto, Japan, #RR036A). The qRT-PCR analysis was conducted on a CFX Connect real-time system (Bio-Rad) by using TB Green Premix Ex Taq II (Tli RNaseH Plus) (Takara, #RR820A). The thermal cycling conditions were as follows: 30 s at 95°C for initial denaturation, 49 cycles of 5 s at 95°C, 30 s at 58°C, and 20 s at 72°C for amplification, and finally 10 min at 72°C for extension. GAPDH was applied for an endogenous standard control. Each reaction was run in triplicates. The relative level of plasma exosomal lncRNAs was calculated using the 2^-ΔCt^ method. The detailed sequences of all the primers (Sangon, Shanghai, China) used in this study are presented in **Table 2**.

**Table 2:** The PCR primer sequences for each gene used in this study.

Genes	Sequences (5’ - 3’)
*LINC00265*	F: 5’-GGAAGAGAGACTGACTGGGC-3’
	R: 5’-GTTTCGCTGTCACCCCTCTG-3’
*PCAT18*	F: 5’-AGGAGACAGGCCCCAGATTT-3’
	R: 5’-TGAAGTGCTGGGACAACGTA-3’
*UCA1*	F: 5’-CTCTCCATTGGGTTCACCATTC-3’
	R: 5’-GCGGCAGGTCTTAAGAGATGAG-3’
*SNHG1*	F: 5’-ACGTTGGAACCGAAGAGAGC-3’
	R: 5’-GCAGCTGAATTCCCCAGGAT-3’
*LINC00467*	F: 5’-CAGCACCGATCCCGACATAG-3’
	R: 5’-CTGGCTTCCTGAAGACGATGA-3’
*GAPDH*	F: 5’-GAAGGGCTCATGACCACAGT-3’
	R: 5’-GGATGCAGGGATGATGTTCT-3’

F, stands for forward; R, stands for reverse.

### Stability testing of plasma exosomal lncRNAs

Three experiments were carried out to determine the stability of lncRNAs in plasma exosomes. Firstly, the exosome suspension derived from the plasma of the AML patient was aliquoted into four parts and stood at RT for 0, 12, 24, and 48 h, respectively. Additionally, the exosome suspension was aliquoted into three parts and subjected to freeze-thaw for 0, 4, and 6 cycles at -80°C, respectively. Furthermore, the exosome suspension was aliquoted into two parts and treated without or with RNase A solution (Solarbio, Beijing, China, #R1030) at a final concentration of 2 μg/mL at 37°C for 20 min. After processions of exosome samples following the above protocols, plasma exosomal RNA was isolated by the aforementioned exoRNeasy Midi Kit, and qRT-PCR analyses were used for plasma exosomal lncRNA quantification.

### Statistical analysis

Statistical analysis was conducted using GraphPad Prism (Version 7.00) or SPSS (Version 25.00). For clinical parameter comparisons between groups, the chi-square test was used to compare the difference in categorical data. The distribution characteristics of the data were determined using the Shapiro-Wilk normality test. The homogeneous variance assumptions were performed using the Brown-Forsythe test. The date with normal distribution and homogeneous variances were presented as the mean ± standard deviation (SD). Comparisons between two groups were conducted using unpaired Student’s t-test. The data with non-normal distribution or heterogeneous variances were presented as the median (interquartile range) (IQR). Comparisons between two groups were conducted using the Wilcoxon matched-pairs signed-rank test or Mann-Whitney U test, and comparisons among three or more groups were conducted using the Kruskal-Wallis test. Multiple comparisons included two-stage linear step-up procedure of Benjamini, Krieger and Yekutieli. For the diagnostic power analyses of the plasma exosomal lncRNAs, the area under the curve (AUC), sensitivity, and specificity were calculated using receiver operating characteristic (ROC) analysis. The optimal cut-off points of exosomal lncRNAs were determined with the largest Youden index (sensitivity+specificity-1). Binary logistic regression analysis was used to establish the combination of exosomal lncRNAs for discriminating AML from HD. The two-tailed p-value<0.05 was considered statistically significant.

## Results

### Characterization of exosomes in plasma

**Figure 1:**
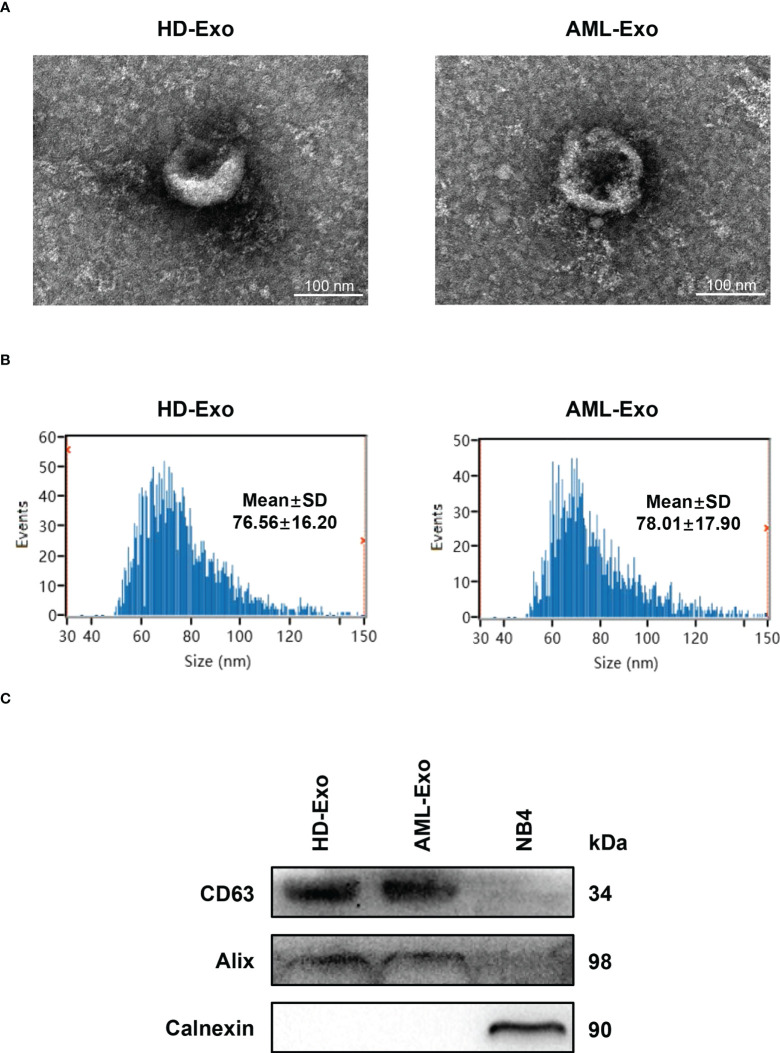
Characterization of Exosomes in Plasma. **(A)** Visualization of the morphologies of plasma exosomes isolated from the healthy donor (HD-Exo) and the newly diagnosed AML patient (AML-Exo) by transmission electron microscopy (TEM). **(B)** Measurement of the size distribution of HD-Exo and AML-Exo by nanoparticle tracking analysis (NTA). SD, standard deviation. **(C)** Western blot analysis of the level of exosome markers (CD63 and Alix) and a non-exosome marker (Calnexin) in HD-Exo, AML-Exo, and NB4 cell lysates.

We first verified whether exosomes were successfully extracted from the plasma specimens of the acute myeloid leukemia (AML) patient and the healthy donor (HD) by a membrane-based affinity binding kit. The purified exosomes were vesicles with a double-layer membrane structure under transmission electron microscopy (TEM) (**Figure 1A**). Nanoparticle tracking analysis (NTA) revealed that the size distribution of exosomes was approximately 60-100 nm in diameter (**Figure 1B**). Besides, the presence of exosome markers (CD63 and Alix) in the exosomes, but not in the lysates of AML cell line NB4, was confirmed by western blotting. Meanwhile, Calnexin as a non-exosome marker only exists in the cell lysates (**Figure 1C**). These data indicated that exosomes are successfully separated from plasma.

### Identification of the dysregulated plasma exosomal lncRNAs in AML

**Figure 2:**
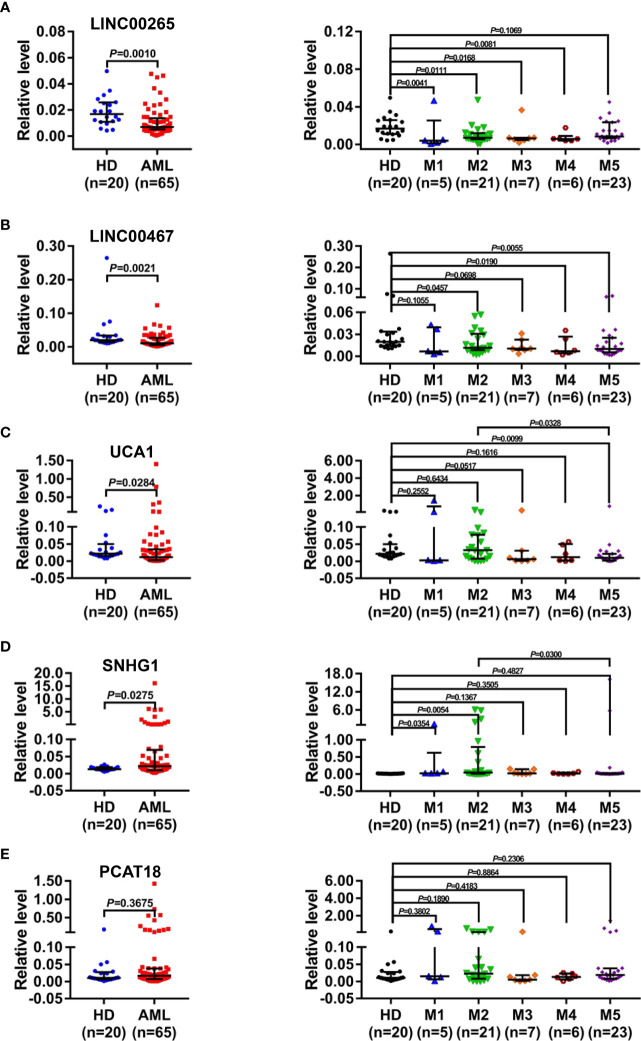
Identification of the Dysregulated Plasma Exosomal LncRNAs in AML. **(A-E, left)** qRT-PCR analysis of the level of plasma exosomal lncRNA LINC00265 (fold change=0.41, *P* = 0.0010), LINC00467 (fold change=0.53, *P* = 0.0021), UCA1 (fold change = 0.53, *P* = 0.0284), SNHG1 (fold change = 1.67, *P* = 0.0275), and PCAT18 in healthy donors (HD) (n = 20) and newly diagnosed AML patients (n = 65). The Shapiro-Wilk normality test was performed to test normality, and then the Mann-Whitney U test was used to compare differences between two groups and determine *P* values. **(A-E**, right**)** Comparison of the level of five plasma exosomal lncRNAs in HD (n = 20), patients with the M1 (n = 5), M2 (n = 21), M3 (n = 7), M4 (n = 6), and M5 (n = 23) subtype. M1-M5 are different French-American-British subtypes of AML (M1: Acute myeloblastic leukemia without maturation; M2: Acute myeloblastic leukemia with maturation; M3: Acute promyelocytic leukemia; M4: Acute myelomonocytic leukemia; M5: Acute monoblastic or monocytic leukemia). The Shapiro-Wilk normality test and Brown-Forsythe test were performed to test normality and homogeneity of variance, and then the Kruskal-Wallis test was used to compare differences among six groups and determine *P* values. Multiple comparisons included two-stage linear step-up procedure of Benjamini, Krieger and Yekutieli. GAPDH was used as the internal control. Data were presented as the median (interquartile range) (IQR).

Next, to define the promising plasma exosomal long non-coding RNAs (lncRNAs) as cell-free biomarkers for AML, a total of five lncRNAs, which were differentially expressed in bone marrow or peripheral blood samples from AML patients and played important roles in leukemogenesis and development (17–21), were selected as candidates. The level of the selected lncRNAs in the plasma exosomes isolated from newly diagnosed AML patients (n=65) and HD (n=20) was analyzed by qRT-PCR. The results showed significant downregulation of the exosomal LINC00265 level in AML patient specimens compared with HD specimens (*P* < 0.05, **Figure 2A**). Particularly, the exosomal LINC00265 downregulation appeared to occur preferentially in the patients with M1, M2, M3, and M4 French-American-British (FAB) subtypes. The exosomal LINC00467 level was decreased in AML cases, especially in the M2, M4, and M5 FAB subtypes (all, *P* < 0.05, **Figure 2B**). Meanwhile, the reduction of the exosomal UCA1 level was also observed in the AML group, especially in the M5 subgroup (all, *P* < 0.05, **Figure 2C**). Of note, the relative expression of exosomal UCA1 was lower in the patients with the M5 subtype than those with the M2 subtype. Additionally, exosomal SNHG1 was highly expressed in AML cases, including M1 and M2 subtypes (all, *P* < 0.05, **Figure 2D**). Interestingly, the M2-subtype patients had a relatively higher exosomal SNHG1 expression than the M5-subtype patients. However, no significant difference in the exosomal PCAT18 level was measured between AML patients and HD (all, *P* > 0.05, **Figure 2E**). These data identified the downregulated plasma exosomal lncRNAs (LINC00265, LINC00467, and UCA1) and the upregulated plasma exosomal lncRNA (SNHG1) in AML patients in comparison with HD, and showed that the dysregulations of these exosomal lncRNAs also exist in the patients with specific FAB subtypes.

### Diagnostic efficiency of the plasma exosomal lncRNAs for AML

**Figure 3:**
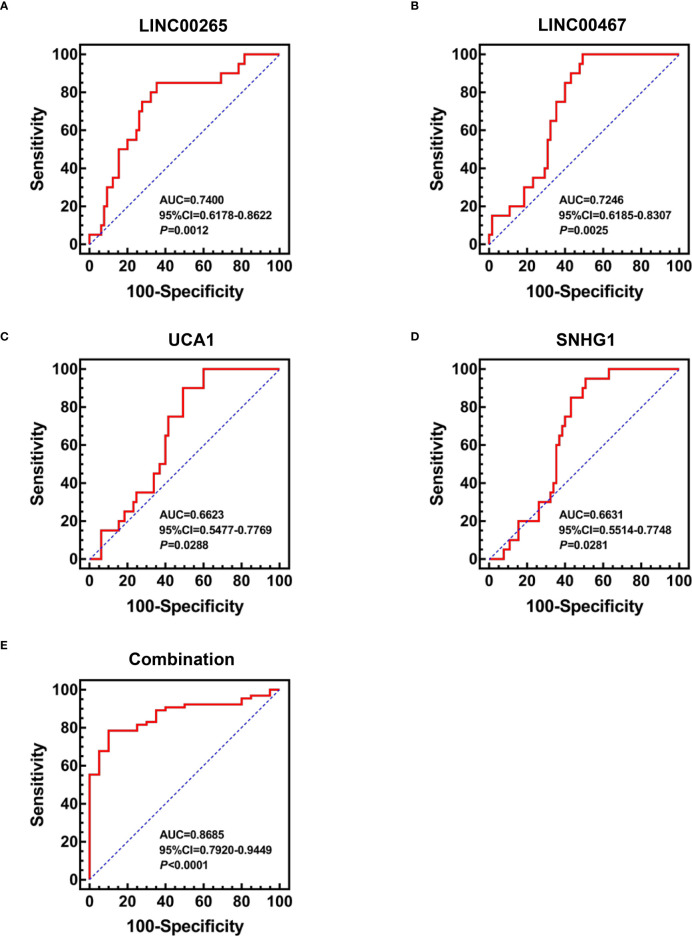
Diagnostic Efficiency of the Plasma Exosomal LncRNAs for AML. **(A-D)** ROC analysis of the individual diagnostic efficiency of four plasma exosomal lncRNAs (LINC00265, LINC00467, UCA1, and SNHG1) in distinguishing AML patients (n = 65) from HD (n = 20). **(E)** ROC analysis and Binary logistic regression analysis of the combined diagnostic efficiency of four plasma exosomal lncRNAs (LINC00265, LINC00467, UCA1, and SNHG1) in distinguishing AML patients (n = 65) from HD (n = 20). AUC: area under the curve; CI: confidence interval.

Based on the aforementioned dysregulations, the diagnostic efficiency of plasma exosomal LINC00265, LINC00467, UCA1, and SNHG1 in differentiating AML patients (n=65) from HD (n=20) was evaluated by receiver operating characteristic (ROC) analysis. As a result, exosomal LINC00265 displayed a relatively high area under the curve (AUC) value of 0.7400 and the cut-off value was at 0.0090 (sensitivity, 85.0%; specificity, 64.6%) (*P* < 0.01, **Figure 3A**). The AUC value of exosomal LINC00467 was 0.7246 and the cut-off value was at 0.0104 (sensitivity, 100.0%; specificity, 50.8%) (*P* < 0.01, **Figure 3B**). Meanwhile, exosomal UCA1 exhibited an AUC value of 0.6623 and the cut-off value was at 0.0122 (sensitivity, 90.0%; specificity, 50.8%) (*P* < 0.05, **Figure 3C**) and exosomal SNHG1 possessed an AUC value of 0.6631 and the cut-off value was at 0.0227 (sensitivity, 95.0%; specificity, 49.2%) (*P* < 0.05, **Figure 3D**). Furthermore, the combination of these four exosomal lncRNAs provided the most powerful performance with an AUC of 0.8685 (*P* < 0.001, **Figure 3E**). These data provided evidence that individual exosomal LINC00265, LINC00467, UCA1, or SNHG1 has a capability for discriminating AML patients from HD, and the combination of these four exosomal lncRNAs displayed the most powerful diagnostic accuracy.

### Correlation of the plasma exosomal lncRNA expression with clinical characteristics of AML patients

The correlation between the expression of plasma exosomal LINC00265, LINC00467, UCA1, and SNHG1 at diagnosis and clinical characteristics of AML patients including gender, age, white blood cell (WBC) count, platelet (PLT) count, hemoglobin (Hb) level, lactate dehydrogenase (LDH) level, percentage of bone marrow blasts, FAB subtype, risk classification, remission response to chemotherapy, and disease recurrence was summarized in **Table 3**. The low expression of exosomal LINC00265 and LINC00467 was associated with young age, while the low expression of exosomal LINC00467 and UCA1 was related to high WBC count (all, *P* < 0.05). However, the expression of these three exosomal lncRNAs was not associated with gender, PLT count, Hb level, LDH level, percentage of bone marrow blasts, FAB subtype, risk classification, remission response to chemotherapy, and disease recurrence (all, *P* > 0.05). Moreover, there was no statistical correlation between the exosomal SNHG1 level and clinical parameters (all, *P* > 0.05). These observations suggested that the low expression of exosomal LINC00265, LINC00467, and UCA1, but not SNHG1, is associated with young age or high WBC count of AML patients.

**Table 3:** Correlation between the plasma exosomal lncRNA level and clinical characteristics of patients with AML (n = 65) [median (IQR)].

Categories	Cases	LINC00265	*P*-value	LINC00467	*P*-value	UCA1	*P*-value	SNHG1	*P*-value
Gender									
Female	34	0.0069 (0.0055-0.0113)	0.8679	0.0089 (0.0057-0.0151)	0.1147	0.0101 (0.0023-0.0312)	0.5526	0.0214 (0.0093-0.0556)	0.5904
Male	31	0.0069 (0.0048-0.0163)		0.0143 (0.0066-0.0352)		0.0141 (0.0025-0.0483)		0.0217 (0.0113-0.0780)	
Age (years)									
<60	47	0.0064 (0.0047-0.0102)	0.0272	0.0088 (0.0056-0.0174)	0.0206	0.0109 (0.0024-0.0327)	0.6160	0.0232 (0.0095-0.0522)	0.4696
≥60	18	0.0109 (0.0065-0.0197)		0.0194 (0.0096-0.0378)		0.0150 (0.0044-0.0626)		0.0201 (0.0132-0.3011)	
Peripheral blood									
WBC count (×10^9^/L)									
<10	32	0.0071 (0.0057-0.0159)	0.4054	0.0118 (0.0087-0.0308)	0.0152	0.0194 (0.0065-0.0546)	0.0449	0.0244 (0.0157-0.1483)	0.0513
≥10	33	0.0066 (0.0041-0.0114)		0.0068 (0.0034-0.0170)		0.0070 (0.0015-0.0271)		0.0135 (0.0089-0.0486)	
PLT count (×10^9^/L)									
<100	59	0.0069 (0.0051-0.0115)	0.3661	0.0100 (0.0056-0.0252)	0.0919	0.0103 (0.0023-0.0327)	0.2694	0.0217 (0.0097-0.0655)	0.8843
≥100	6	0.0128 (0.0048-0.0289)		0.0204 (0.0127-0.0439)		0.0248 (0.0135-0.0408)		0.0254 (0.0091-1.4860)	
Hb level (g/L)									
<100	53	0.0069 (0.0053-0.0114)	0.7930	0.0104 (0.0057-0.0304)	0.5643	0.0156 (0.0026-0.0373)	0.3704	0.0207 (0.0105-0.0924)	0.6929
≥100	12	0.0084 (0.0024-0.0183)		0.0100 (0.0059-0.0221)		0.0069 (0.0019-0.0336)		0.0237 (0.0078-0.0449)	
LDH level (U/L)									
<250	7	0.0086 (0.0041-0.0280)	0.3661	0.0195 (0.0117-0.0352)	0.1606	0.0159 (0.0027-0.0573)	0.4842	0.0283 (0.0125-0.0655)	0.6801
≥250	54	0.0067 (0.0051-0.0118)		0.0095 (0.0055-0.0260)		0.0106 (0.0025-0.0350)		0.0225 (0.0102-0.0673)	
Unknown	4								
Bone marrow blasts (%)									
<50	22	0.0070 (0.0059-0.0113)	0.6523	0.0139 (0.0085-0.0287)	0.2914	0.0109 (0.0046-0.0434)	0.5586	0.0234 (0.0083-0.0573)	0.9372
≥50	43	0.0069 (0.0047-0.0148)		0.0091 (0.0050-0.0242)		0.0141 (0.0023-0.0322)		0.0207 (0.0104-0.0780)	
FAB subtype									
M0	1	0.0069 (-)	0.8078	0.0110 (-)	0.9562	0.0162 (-)	0.1882	0.0161 (-)	0.8936
M1	5	0.0039 (0.0010-0.0255)		0.0066 (0.0040-0.0396)		0.0025 (0.0012-0.7580)		0.0240 (0.0197-0.6232)	
M2	21	0.0071 (0.0055-0.0121)		0.0117 (0.0084-0.0307)		0.0322 (0.0074-0.0776)		0.0448 (0.0139-0.7930)	
M3	7	0.0064 (0.0047-0.0072)		0.0105 (0.0088-0.0227)		0.0061 (0.0018-0.0308)		0.0235 (0.0087-0.1410)	
M4	6	0.0059 (0.0041-0.0090)		0.0068 (0.0040-0.0268)		0.0120 (0.0020-0.0503)		0.0183 (0.0096-0.0427)	
M5	23	0.0086 (0.0066-0.0235)		0.0100 (0.0056-0.0252)		0.0099 (0.0024-0.0214)		0.0124 (0.0081-0.0283)	
Unclassified	2								
Risk classification									
Favorable	16	0.0076 (0.0048-0.0221)	0.5102	0.0158 (0.0042-0.0315)	0.8004	0.0187 (0.0063-0.0484)	0.6207	0.0238 (0.0162-0.1312)	0.2481
Intermediate	25	0.0063 (0.0044-0.0118)		0.0100 (0.0061-0.0193)		0.0156 (0.0021-0.0450)		0.0249 (0.0120-0.0951)	
Adverse	13	0.0078 (0.0053-0.0147)		0.0088 (0.0050-0.0338)		0.0109 (0.0025-0.0282)		0.0113 (0.0078-0.0402)	
Unclassified	11								
Remission response to chemotherapy									
NR/PR	5	0.0066 (0.0059-0.0201)	0.5597	0.0035 (0.0017-0.0370)	0.2202	0.0026 (0.0007-0.0152)	0.0513	0.0124 (0.0090-0.0342)	0.1466
CR	40	0.0065 (0.0047-0.0112)		0.0111 (0.0070-0.0308)		0.0194 (0.0059-0.0484)		0.0240 (0.0133-0.1036)	
Unknown	20								
Disease recurrence									
No	37	0.0066 (0.0049-0.0139)	0.2982	0.0105 (0.0062-0.0275)	0.6478	0.0175 (0.0059-0.0484)	0.6478	0.0240 (0.0113-0.1265)	0.4184
Yes	2	0.0049 (0.0041-0.0057)		0.0641 (0.0046-0.1236)		0.0141 (0.0027-0.0254)		0.0161 (0.0125-0.0196)	
Unknown	1								

FAB subtype, French-American-British subtype, a classification of acute leukemia produced by three-nation joint collaboration; NR, non-remission; PR, partial remission; CR, complete remission. The Shapiro-Wilk normality test and Brown-Forsythe test were performed to test normality and homogeneity of variance. The Mann-Whitney U test was used to compare differences between two groups and determine P values. The Kruskal-Wallis test was used to compare differences among three or more groups and determine P values. P < 0.05 means statistically significant.

### Treatment monitoring capability of the plasma exosomal lncRNAs for AML

**Figure 4:**
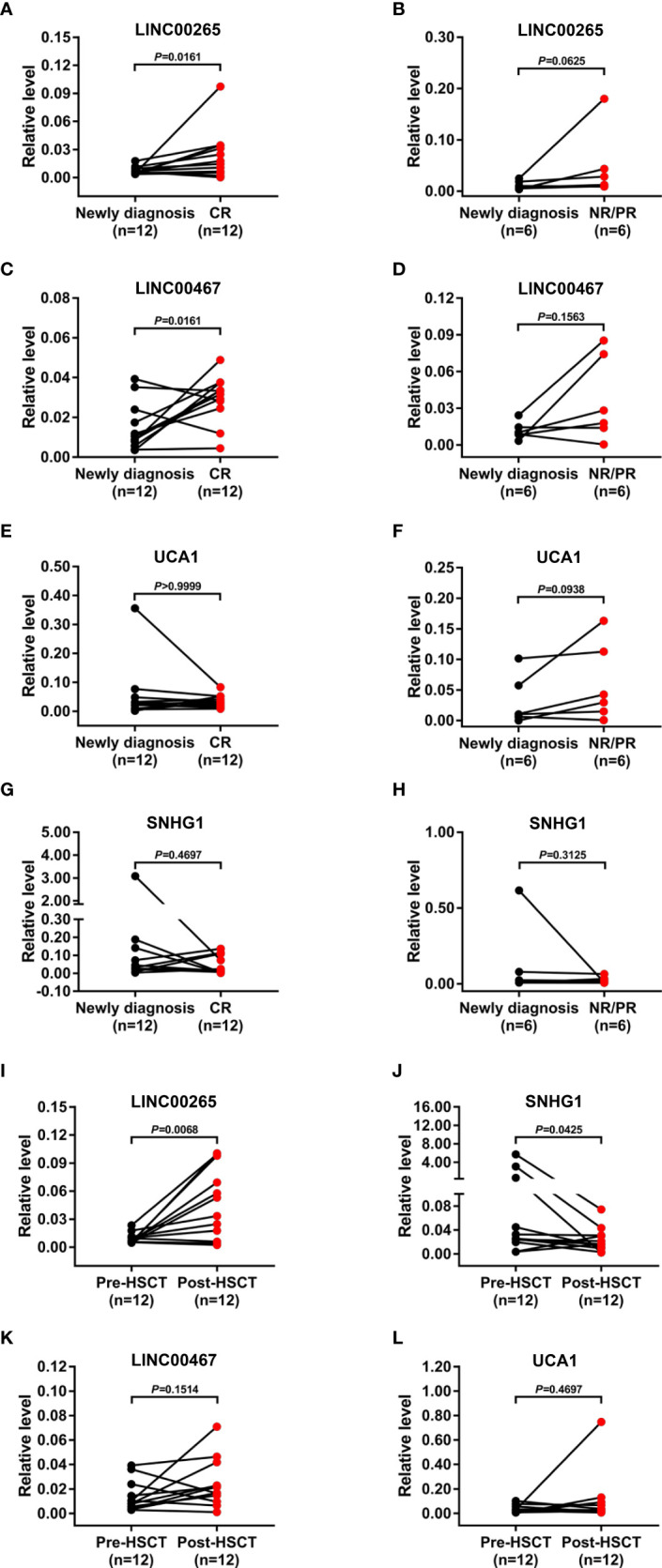
Treatment Monitoring Capability of the Plasma Exosomal LncRNAs for AML. **(A, C, E, G)** qRT-PCR analysis of the level of four plasma exosomal lncRNAs (LINC00265, LINC00467, UCA1, and SNHG1) in the paired samples from AML patients (n = 12) at newly diagnosis and first complete remission (CR) stage. **(B, D, F, H)** qRT-PCR analysis of the level of four plasma exosomal lncRNAs (LINC00265, LINC00467, UCA1, and SNHG1) in the paired samples from AML patients (n = 6) at newly diagnosis and at non-remission (NR) or partial remission (PR) stage. **(I-L)** qRT-PCR analysis of the level of four plasma exosomal lncRNAs (LINC00265, SNHG1, LINC00467, and UCA1) in the paired samples from AML patients (n = 12) before allogeneic hematopoietic stem cell transplantation (allo-HSCT) (Pre-HSCT) and after allo-HSCT (Post-HSCT). The Shapiro-Wilk normality test was performed to test normality. The Wilcoxon matched-pairs signed-rank test was used to compare differences between two groups and determine *P* values. GAPDH was used as the internal control. Data were presented as the median (IQR).

We further assessed the treatment monitoring power of plasma exosomal LINC00265, LINC00467, UCA1, and SNHG1 for AML. The relevance of the expression of these exosomal lncRNAs to the remission status of AML patients after the standard induction chemotherapy was first explored. The level of both exosomal LINC00265 and exosomal LINC00467 was elevated in the samples at first complete remission (CR) (all, *P* < 0.05, **Figures 4A, C**), but not at non-remission (NR) or partial remission (PR) (all, *P* > 0.05, **Figures 4B, D**), compared with the paired samples at newly diagnosis. However, the exosomal UCA1 or SNHG1 expression remained unchanged in the samples at the CR, NR, and PR stages (all, *P* > 0.05, **Figures 4E-H**). Subsequently, the relationship between the expression of these four exosomal lncRNAs and the allogeneic hematopoietic stem cell transplantation (allo-HSCT) treatment was analyzed. The data showed upregulation of the exosomal LINC00265 level and downregulation of the exosomal SNHG1 level upon the allo-HSCT treatment (all, *P* < 0.05, **Figures 4I, J**). Nevertheless, there was no significant change in the exosomal LINC00467 or UCA1 expression between the samples before and after allo-HSCT (all, *P* > 0.05, **Figures 4K, L**). These results indicated that the increased expression of exosomal LINC00265 and LINC00467 is associated with the CR but not NR or PR status of AML patients, while the increased expression of exosomal LINC00265 and the decreased expression of SNHG1 is associated with the allo-HSCT treatment.

### Stability evaluation of the dysregulated lncRNAs in plasma exosomes

**Figure 5:**
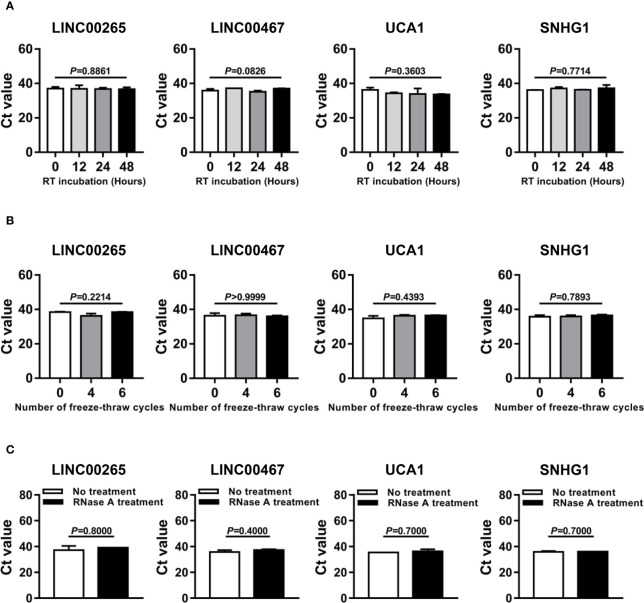
Stability Evaluation of the Dysregulated LncRNAs in Plasma Exosomes. **(A)** Aliquots of plasma exosome suspensions isolated from the AML patient were incubated at room temperature (RT) for 0, 12, 24, and 48 h, respectively. qRT-PCR analysis of the threshold cycle (Ct) value of four plasma exosomal lncRNAs (LINC00265, LINC00467, UCA1, and SNHG1) in the pretreated suspensions. The Shapiro-Wilk normality test and Brown-Forsythe test were performed to test normality and homogeneity of variance, and then the Kruskal-Wallis test was used to compare differences among four groups and determine *P* values. **(B)** Aliquots of plasma exosome suspensions isolated from the AML patient were subjected to freeze-thaw for 0, 4, and 6 cycles at -80 °C, respectively. qRT-PCR analysis of the Ct value of four plasma exosomal lncRNAs (LINC00265, LINC00467, UCA1, and SNHG1) in the pretreated suspensions. The Shapiro-Wilk normality test and Brown-Forsythe test were performed to test normality and homogeneity of variance, and then the Kruskal-Wallis test was used to compare differences among three groups and determine *P* values. **(C)** Aliquots of plasma exosome suspensions isolated from the AML patient were treated without or with RNase A solution (2 μg/ml) at 37 °C for 20 min. qRT-PCR analysis of the Ct value of four plasma exosomal lncRNAs (LINC00265, LINC00467, UCA1, and SNHG1) in the pretreated suspensions. The Shapiro-Wilk normality test was performed to test normality, and then the Mann-Whitney U test was used to compare differences between two groups and determine *P* values. Data were presented as the median (IQR).

Given that better stability is an essential prerequisite for tumor biomarkers, we sought to assess the stability of LINC00265, LINC00467, UCA1, and SNHG1 in the isolated plasma exosomes. Firstly, the exosome suspensions were subjected to room temperature incubation for 0, 12, 24, and 48 h. As anticipated, the expression level of exosomal LINC00265, LINC00467, UCA1, and SNHG1 remained unchanged following prolonged exposure at room temperature (all, *P* > 0.05, **Figure 5A**). In addition, multiple freeze-thaw cycles of the exosome suspensions made no significant difference in the level of these four exosomal lncRNAs (all, *P* > 0.05, **Figure 5B**). Finally, the exosome suspensions were treated with RNase A and this treatment had little effect on the stability of exosomal lncRNAs (all, *P* > 0.05, **Figure 5C**). These results demonstrated that the candidate lncRNAs are high stability in plasma exosomes.

## Discussion

Exosomes are a class of extracellular vesicles that deliver specific combinations of nucleic acids, proteins, and lipids, facilitating tumorigenesis and development (35). Recently, compelling evidence has corroborated that long non-coding RNAs (lncRNAs) can stably exist in exosomes, and be examined for diagnosis, prediction, and surveillance in various cancers (8). However, there is limited knowledge on the potential clinical utility of exosomal lncRNAs in acute myeloid leukemia (AML). Herein, our data proved that plasma exosomal LINC00265, LINC00467, UCA1, and SNHG1 might act as promising cell-free biomarkers for diagnosis and treatment monitoring of AML.

In the present study, plasma exosomes, which were bilayer vesicles with a diameter of approximately 60-100 nm containing the exosome markers (CD63 and Alix), were first efficiently extracted from the AML patient and the healthy donor (HD) by a membrane-based affinity binding kit. Nowadays, multiple strategies have been developed for exosome isolation including ultracentrifugation, size-based separation, capture-based separation, acoustic-based separation, polymer precipitation methods, and so on (12, 36). Although considered as the gold standard, the process of ultracentrifugation is time-consuming and highly instrument-dependent (36). Given the advantages of a sample, high efficiency, and reproducibility, the membrane-based affinity binding technique was extensively used for exosome purification and easily adapted to clinical laboratory workflows (37, 38). Next, we sought to identify the aberrantly expressed lncRNAs in the plasma exosomes of AML patients. There were five lncRNAs included in our initial study due to their abnormal expression in bone marrow or peripheral blood samples of AML patients and crucial roles in leukemogenesis and development (17–21). Additionally, these five lncRNAs have not been reported relating to AML exosomes. The results showed that the plasma exosomal lncRNAs LINC00265, LINC00467, and UCA1 were downregulated while SNHG1 was upregulated in AML patients in comparison with those in HD. Meanwhile, the dysregulations were observed in the patients with specific French-American-British (FAB) subtypes. It has been well known that the FAB classification of AML is mainly based on the direction of differentiation along one or more cell lines and the maturation degree of the cells in the bone marrow and peripheral blood, which can reflect the progression of AML (31). Therefore, our findings suggest that the level of these four plasma exosomal lncRNAs may be associated with the progression of AML, making them particularly attractive as biomarkers for AML. Up to now, sequencing or microarray analysis has been broadly applied for the exploration of cancer biomarkers. Lin et al. (39) performed RNA sequencing to screen early gastric cancer (EGC)-specific exosomal lncRNAs. In addition, a study reported (40) that microarray analysis was used to explore the differential exosomal lncRNAs in colorectal cancer (CRC). Although four dysregulated exosomal lncRNAs were identified in our study, it is necessary for us to explore more exosomal lncRNAs by high-throughput technologies as potential biomarkers for AML in the future.

In view of the aforementioned dysregulations, we further investigated the clinical significance of these four plasma exosomal lncRNAs (LINC00265, LINC00467, UCA1, and SNHG1) for AML. Firstly, the individual diagnostic efficiency of these exosomal lncRNAs for newly diagnosed AML by receiver operating characteristic (ROC) analysis was assessed. Our results showed that exosomal LINC00265, LINC00467, UCA1, or SNHG1 had the capability for discriminating AML patients from HD. Interestingly, a present study reported (27) that there was no significant difference in the serum exosomal UCA1 expression between healthy controls and newly diagnosed multiple myeloma patients, perhaps exosomal UCA1 exhibits relatively high specificity for AML diagnosis. Recently, Zhang et al. (41) identified a promising diagnostic panel based on three exosomal lncRNAs (PCAT-1, UBC1, and SNHG16) to differentiate bladder cancer cases from healthy controls with excellent accuracy. Our previous study showed that the combination of two exosomal lncRNAs (TBILA and AGAP2-AS1) failed to provide better results than individual exosomal lncRNAs in the detection of non-small-cell lung cancer (NSCLC) with pathologic subtypes and early-stage (29). Based on the above studies, we also analyzed the combined diagnostic efficacy of exosomal LINC00265, LINC00467, UCA1, and SNHG1. Intriguingly, this combination exhibited the highest discriminatory capacity for AML patients from HD. These observations suggest that these four plasma exosomal lncRNAs, alone or in combination, hold promise as diagnostic biomarkers for AML. In fact, exosomes are enriched in other kinds of non-coding RNA (ncRNA) molecules such as circular RNA (circRNA), tRNA-derived small RNA (tsRNA), ribosomal RNA, and transfer RNA (7, 42, 43). Pan et al. (44) identified serum exosomal circ-0004771 as a novel diagnostic biomarker of CRC. Besides, four tsRNAs were reported to be increased expressed in plasma exosomes from liver cancer patients (42). Thus, further study deserves to be performed to investigate the diagnostic performance and even other clinical significance of other exosomal ncRNAs for AML. Subsequently, the association between the expression of exosomal LINC00265, LINC00467, UCA1, and SNHG1 at diagnosis and clinical characteristics of AML patients was analyzed. The low expression of exosomal LINC00265 and LINC00467 was associated with young age, while the low expression of exosomal LINC00467 and UCA1 was related to high WBC count. These results showed the expression of exosomal LINC00265, LINC00467, and UCA1 was closely correlated with the progression of AML. Of note, there was no statistical correlation of the expression of these four exosomal lncRNAs at diagnosis with remission response to chemotherapy, disease recurrence, and risk classification, suggesting their limited value in AML therapeutic effect and prognostic prediction.

Since plasma-derived exosomes are widely known to be abundantly generated by stressed cells (45), it seems conceivable that the level of exosomal lncRNAs is associated with disease severity and treatment response in AML. Indeed, our results showed that the level of exosomal LINC00265 and LINC00467 was elevated in the samples at first complete remission (CR), but not at the non-remission (NR) or partial remission (PR), indicating that the exosomal expression of LINC00265 and LINC00467 may be useful in remission assessment in AML patients receiving chemotherapy. Likewise, Hong et al. found significant alterations in the exosomal protein level after AML patients underwent chemotherapy. Additionally, in some patients under consolidation therapy who subsequently relapsed, the expression of exosomal proteins was upregulated (46). Thus, it is imperative for us to exploit the roles of exosomal lncRNAs in monitoring AML residual disease and recurrence. Meanwhile, our results revealed that there was upregulation of the exosomal LINC00265 level and downregulation of the exosomal SNHG1 level upon the allogeneic hematopoietic stem cell transplantation (allo-HSCT) treatment, suggesting that the expression of exosomal LINC00265 and SNHG1 may reflect the tumor burden of AML patients and can be applied for estimating the therapeutic efficacy of allo-HSCT. These findings indicated that the expression of exosomal LINC00265, LINC00467, and SNHG1, but not UCA1, was sensitive to the standard induction chemotherapy or allo-HSCT treatment, showing the potential of these three exosomal lncRNAs as indicators of disease activity during therapy. It is generally accepted that the assessment of AML treatment responses relies on comprehensive cellular analyses of morphology, immunophenotype, cytogenetics, and molecular genetics, which is made difficult by the rapid clearance of circulating leukemic blasts during therapy. In contrast, exosomal lncRNAs serve as cell-free biomarkers, representing an important advance in cancer liquid biopsy as the detection of their expression is less invasive, less expensive, and provides real-time insights into tumor status. Notably, several patients displayed unchanged or even decreased level of LINC00265 and LINC00467 at CR or after allo-HSCT, which might be due to the high heterogeneity of AML. Accordingly, treatment monitoring of these exceptional patients should depend on a combination of exosomal lncRNA detection and cellular analyses. In future studies, we will explore more effective cell-free biomarkers for estimating the therapeutic efficacy of AML patients. Given that better stability is an essential requirement for tumor biomarkers, the stability testing of LINC00265, LINC00467, UCA1, and SNHG1 in the isolated plasma exosomes was performed. The level of these four exosomal lncRNAs was not significantly influenced following prolonged exposure to 48 h at room temperature, multiple freeze-thaw cycles, or RNase A treatment. Recently, Li et al. (40) observed no significant change in the exosomal lncRNA expression when the exosome suspensions were incubated with prolonged exposure to room temperature or treated with RNase A. In another study, exosomes could protect contained lncRNA HOTTIP from degradation by prolonged exposure or multiple freeze-thaw cycles (47). Results from our experiments are consistent with the above reports, which reveal that the candidate lncRNAs in our study have good stability in plasma exosomes and are worthy of further research. Besides, the existing study also documented that serum exosomal lncRNA CRNDE-h remained stable after the exosome samples were subjected to acid-base incubation or stored at -80°C for a long time (48). Therefore, a deeper exploration of the stability of LINC00265, LINC00467, UCA1, and SNHG1 in plasma exosomes under other harsh conditions is required to improve the clinical potential of these molecules. Taken together, these preliminary data suggest that continuous monitoring of exosomal lncRNAs may be important in the search for new tests for AML diagnosis, disease progression, and response to treatment.

Indeed, we are also aware of the potential limitations of this study. Firstly, the sample size needs to enlarge, and the clinical significance of these four exosomal lncRNAs in AML needs to be validated in prospective and multicenter studies. Secondly, the specificity of these exosomal lncRNAs for AML diagnosis is required to confirm by examining those in patients with other cancers. Additionally, the prognostic value of exosomal lncRNAs deserved to be investigated. Furthermore, more researches are worthy to illustrate the function and mechanisms of exosomal lncRNAs in AML.

## Data availability statement

The original contributions presented in the study are included in the article/supplementary material. Further inquiries can be directed to the corresponding authors.

## Ethics statement

The studies involving human participants were reviewed and approved by the ethics committee of Chongqing Medical University. The patients/participants provided their written informed consent to participate in this study.

## Author contributions

LZ, QX, and CL initiated the work and designed the experiments. QX and CL performed the experiments and wrote the manuscript. MP, JR, and YJ contributed techniques and commented on the manuscript. YT analyzed the data. LL and JH contributed analytic tools. ZSY, ZLY, and JW provided clinical assistance. JY and MS assisted with revising the manuscript. All authors contributed to the article and approved the submitted version.

## Funding

This work was supported by the National Natural Science Foundation of China (NSFC81873973 and NSFC82072353), the Natural Science Foundation of CQ CSTC (cstc2021jcyj-msxmX0363), and Chongqing medical scientific research project (Joint project of Chongqing Health Commission and Science and Technology Bureau) (2020MSXM074).

## Acknowledgments

The authors would like to acknowledge the Key Laboratory of Laboratory Medical Diagnostics Designated by the Ministry of Education, School of Laboratory Medicine (Chongqing Medical University, Chongqing, China) for providing the space and equipment for conducting the experiments.

## Conflict of interest

The authors declare that the research was conducted in the absence of any commercial or financial relationships that could be construed as a potential conflict of interest.

## Publisher’s note

All claims expressed in this article are solely those of the authors and do not necessarily represent those of their affiliated organizations, or those of the publisher, the editors and the reviewers. Any product that may be evaluated in this article, or claim that may be made by its manufacturer, is not guaranteed or endorsed by the publisher.
